# Lactic Acid Bacteria and Their Bacteriocins: Classification, Biosynthesis and Applications against Uropathogens: A Mini-Review

**DOI:** 10.3390/molecules22081255

**Published:** 2017-07-26

**Authors:** Mduduzi Paul Mokoena

**Affiliations:** Discipline of Microbiology, School of Life Sciences, College of Agriculture, Engineering and Science, University of KwaZulu-Natal, Westville Campus, Private Bag X54001, Durban 4000, South Africa; mokoenap@ukzn.ac.za; Tel.: + 27-31-260-7405

**Keywords:** bacteriocins, lactic acid bacteria, uropathogens

## Abstract

Several lactic acid bacteria (LAB) isolates from the *Lactobacillus* genera have been applied in food preservation, partly due to their antimicrobial properties. Their application in the control of human pathogens holds promise provided appropriate strains are scientifically chosen and a suitable mode of delivery is utilized. Urinary tract infection (UTI) is a global problem, affecting mainly diabetic patients and women. Many uropathogens are developing resistance to commonly used antibiotics. There is a need for more research on the ability of LAB to inhibit uropathogens, with a view to apply them in clinical settings, while adhering to strict selection guidelines in the choice of candidate LAB. While several studies have indicated the ability of LAB to elicit inhibitory activities against uropathogens in vitro, more in vivo and clinical trials are essential to validate the efficacy of LAB in the treatment and prevention of UTI. The emerging applications of LAB such as in adjuvant therapy, oral vaccine development, and as purveyors of bioprotective agents, are relevant in infection prevention and amelioration. Therefore, this review explores the potential of LAB isolates and their bacteriocins to control uropathogens, with a view to limit clinical use of antibiotics.

## 1. Introduction

Lactic acid bacteria (LAB) are a group of Gram-positive, non-spore forming, cocci or rods, catalase-negative, and fastidious organisms, with high tolerance for low pH [1,2,3]. LAB are among the most important microbes which are used in food fermentations, as well as in enhancing taste and texture in fermented food products [1,4]. They are characterized by the production of lactic acid as the main product from glucose and growth inhibition substances such as bacteriocins, hydrogen peroxide, diacyls, etc. which prevent the proliferation of food spoilage bacteria and pathogens [2,5]. Some bacilli such as bifidobacteria also form lactic acid as a major end product. LAB are grouped into the *Clostridium* branch of Gram-positive bacteria which is related to bacilli, whereas *Bifidobacterium* belongs to Actinomycetes. The DNA of LAB has a low G + C content [6].

Lactic acid bacteria ferment carbohydrates to obtain energy, using endogenous carbon sources as the final electron acceptor instead of oxygen. They are aerotolerant, and are protected against oxygen by-products such as hydrogen peroxide by peroxidases. LAB are usually non-motile, and cell division occurs in one plane, except in pediococci [6]. Phenotypic methods have been most commonly used for the identification of LAB, but more recently, molecular techniques such as 16S rDNA sequencing have been developed, enabling a more consistent and accurate identification of individual strains [7]. Other promising identification tools include partial *rRNA* gene sequencing using the polymerase chain reaction, and the soluble protein patterns. The growth optimum for LAB is at pH 5.5–5.8, and these microorganisms have complex nutritional requirements for amino acids, peptides, nucleotide bases, vitamins, minerals, fatty acids and carbohydrates [7]. They are categorized into homofermentative and heterofermentative microorganisms, based on the products of the fermented carbohydrates. Homofermentative LAB mainly produce lactic acid from sugars, whereas heterofermentative LAB produce lactic acid, acetic acid or alcohol and carbon dioxide [8,9,10]. In addition, some species of LAB produce antimicrobial peptides known as bacteriocins. To date, several LAB isolates from the *Lactobacillus* genus and their bacteriocins have been applied in food preservation and in the control of human pathogens [11].

Several authors have documented the ability of various LAB to inhibit growth of pathogenic microorganisms, their ability to degrade mycotoxins, their probiotic capabilities, as well as antimicrobial activities of cell-free extracts of the LAB isolates from different sources [12,13]. While LAB have been used as starter cultures and bacteriocins as food preservatives, there is a need for more information on the inhibitory potential of LAB against human pathogens such as uropathogens, especially in the current era of antibiotic resistant pathogens. It is therefore important to take advantage of the new technologies available to characterize and identify new LAB strains with more potent antimicrobial activities. Hence, this review seeks to explore the properties of LAB isolates and their bacteriocins, and their potential to inhibit uropathogens, thereby ultimately reducing reliance on antibiotics.

## 2. Lactic Acid Bacteria—Classification, Distribution and Sources 

LAB are found in decomposing plant material and fruits, in dairy products, fermented meat and fish, cereals, beets, pickled vegetables, potatoes, sourdough, silages, fermented beverages, juices, sewage and in cavities of humans and animals [6,14,15]. In humans, they particularly inhabit the oral cavity, ileum, colon, and are the dominant organisms in the vagina [13,16]. The LAB group is currently classified in the phylum *Firmicutes*, class *Bacilli*, and order *Latobacillales*. LAB are classified based on cellular morphology, mode of glucose fermentation, range of growth temperature, and sugar utilization patterns [17]. LAB genera include *Lactobacillus*, *Lactococcus, Leuconostoc*, *Pediococcus*, *Streptococcus*, *Aerococcus*, *Alloiococcus*, *Carnobacterium*, *Dolosigranulum*, *Enterococcus*, *Oenococcus*, *Tetragenococcus*, *Vagococcus* and *Weissella* [7,18], with *Lactobacillus* being the largest genus, including more than 100 species that are abundant in carbohydrate-rich substances. 

The majority of *Lactobacillus* species have been isolated from the gastrointestinal tract of humans and animals. The second largest number of *Lactobacillus* species are from vegetables and their fermentation products [13,16], whereas species from the *Leuconostoc* genus are mainly isolated from chilled meats or clinical sources, although they are also obtained from plant material, fermented dairy products and wines [19,20]. Species of the genus *Pediococcus* are associated with spoilage of fermented beverages, especially beers. Although *Lactococcus* species have been isolated from plant material, they are most abundant in dairy products such as sour milk [11]. Members of the genus *Lactobacillus* are also Gram-positive, non-motile and non-sporulating organisms. However, while they are acid-tolerant facultative anaerobes, they can be either homo- or heterofermentative [21].

Due to their health benefits, some LAB are used as probiotics. Probiotics are organisms such as bacteria or yeast that improve human or animal health, and are available in supplements and fermented foods such as yoghurt, or as nutritional supplements that contain live bacteria for building up the intestinal microbiota [4]. For an organism to be a probiotic, it must essentially be non-pathogenic, be generally regarded as safe (GRAS), tolerate low pH, tolerate high concentrations of conjugated and de-conjugated bile salts, be tolerated by the immune system, and should not result in the formation of antibodies [22]. In addition, such an organisms must not confer antibiotic resistance genes to potential pathogens through horizontal gene transfer.

The LAB used as probiotics require a careful safety assessment, and must adhere to strict selection guidelines. Hence, the FDA has established a regulatory authority for probiotics production, manufacturers, labeling and safety of products, despite the GRAS status of these microorganisms. In the FDA, there are four regulatory categories informed by the intended use of the product and each of these has different requirements. These categories are (1) Drug or biological products; (2) Dietary supplements; (3) Food or food ingredient; and (4) Medical food [23,24]. In Europe, probiotic mediated food is not regulated, but microbial feed additives are regulated by a safety assessment of these additives in animals and humans. According to the “Qualified Perception of Safety” (QPS) concept launched by the Scientific Committee on Animal Nutrition in Europe, the species that have adequate safety data are allowed to be marketed without extensive safety testing [25]. The FAO and WHO collaborated to establish guidelines for probiotics in food [26].

## 3. Bacteriocins Produced by Lactic Acid Bacteria

Bacteriocins are a group of potent antimicrobial peptides produced by some microorganisms including LAB, primarily active against closely related organisms, mostly Gram-positive bacteria to gain competitive advantage for nutrients in the environment, and are ribosomally-synthesized as primary metabolites [11,27]. Bacteriocins are small cationic molecules of about 30–60 amino acids, forming amphiphilic helices and stable at 100 °C for 10 min and they differ in spectrum of activity, mode of action, molecular weight (MW), genetic origin and biochemical properties. Bacteriocin-producing LAB strains protect themselves from their own toxins by the expression of a specific immunity protein, encoded in the bacteriocin operon [11].

In fermented foods, LAB display various antimicrobial activities, through production of various metabolites, including lactic acid, hydrogen peroxide, and bacteriocins. Consequently, several bacteriocins with potential industrial applications have been isolated and characterized, and can be used to preserve food when the cold chain falters while heat sensitive food is in transit, although only nisin and pediocin have been approved by the FDA. LAB isolated from homemade fermented vegetables produce antibacterial substances against both Gram-positive and importantly, Gram-negative common foodborne bacterial pathogens. This broad spectrum of inhibition suggests that these LAB strains have a potential as natural biopreservatives in various food products, and may help to combat human pathogens.

Bacteriocins site of action is the bacterial cytoplasmic membrane and they target energized membrane vesicles to disrupt the proton motive force [11]. Their drawback lies in the fact that they inhibit mainly closely related organisms, implying that some of them may inhibit other desirable starter cultures, and may lack activity against Gram-negative pathogens and food spoilers. However, this property may be desirable when LAB are used to target a specific sensitive pathogen. Addition of chelating agents has been effective in rendering Gram-negative bacteria susceptible to bacteriocins [11]. A recent study has established that the use of heterofermentative *Lactobacillus* spp. in the production of Dutch-type cheese products reduces the populations of technologically harmful microorganisms that negatively affect the quality, safety and shelf life of the finished products [28], and may be explored to control human pathogens.

### 3.1. Classes of Bacteriocins Produced by LAB

Bacteriocins are classified based on their primary structures, molecular weights, post-translational modifications and genetic characteristics (Table 1). However, there is no universally adopted classification scheme of LAB. Originally, four classes were recognized, but recently authors have revised them into three [15], although there are slight differences in the description of sub-classes among different authors. Class I bacteriocins are called lantibiotics and are characterized based on their post-translational modification, with nisin and lactocin as representatives. They are extensively post-translationally modified, resulting in the formation of unusual amino acids lanthionine and methyllanthionine [11].

Class II bacteriocins are small (<10 kDa), heat-stable, non-modified peptides cationic, hydrophobic peptides. They are sub-divided into class IIa and Class IIb. Class IIa contains pediocin-like *Listeria* active peptides, and therefore gaining attention in food preservation, with pediocin PA1 and leucocin A as examples [29]. Almost 50 different kinds of class IIa bacteriocins have been isolated from fermented meat, fermented vegetable, dairy products, smoked salmon and the human gastrointestinal tract. Class IIb bacteriocins require the synergistic activity of two complementary peptides in order to exert antimicrobial activity, examples being plantaricin A, and enterocin X [27,30,31]. Although some peptides of this class can individually exert antimicrobial activity, the addition of the complementary peptide greatly enhances this activity. The pair of complementary peptides is active at the nanomolar to picomolar range. Individual Class IIb bacteriocins contain amphiphilic and hydrophobic regions and they are mostly cationic. The genes encoding the two different peptides are genetically closely associated, and encoded in the same operon [32].

Class III bacteriocins consists of large and heat-labile proteins that are more than 30 kDa, with helveticin J as an example [11]. The former class IV bacteriocins consists of large complexes of proteins with other macromolecules, and has been de-established, and member peptides were re-classified as bacteriolysins, i.e. hydrolytic polypeptides; leaving only three classes of bacteriocins, based on the genetic and biochemical characteristics of their members [33].

### 3.2. Biosynthesis and Production of LAB Bacteriocins

The production of bacteriocins depends on the microbial strain and culture conditions. Bacteriocins are ribosomally synthesized peptides, which are initially biologically inactive, and later modified to attain an active state [13,29,33]. Generally, the genes encoding bacteriocin production and immunity are organized in operon clusters. They can be located on mobile genetic elements, such as chromosome in association with transposons or plasmids. Newly synthesized bacteriocins contain an N-terminal leader. They are then modified by proteins or amino acids encoded by the bacteriocin gene cluster before they are exported out of the cell. Some of the modifications include thioether cross-links called lathionines or methyllanthionines formed via the dehydration of serine and threonine residues followed by stereoselective intermolecular addition of cysteins onto the unsaturated amino acids [13,27,29,33].

The major pathways for Class I (lantibiotic) bacteriocin biosynthesis can be described by using the pathway followed by the well-known nisin, although there are slight differences for the non-lantibiotic bacteriocins as they do not need incorporation of unusual amino acids. (1) The *nisA* gene is translated to pre-nisin A peptide; (2) Pre-nisin A is converted into precursor nisin A by the products of the *nisB* and *nisC* genes. Several disulphide bridges are made and some amino acids are converted to unusual ones; (3) The precursor nisin A is exported out of the cell with the aid of the *nisT* and *nisP* while the leader peptide is simultaneously cleaved to obtain the nisin A [36]. Bacteriocin expression is regulated either by external induction factors, usually secreted by the producer strain itself, or it can be constitutive while bacteriocin biosynthesis depends on environmental conditions such as temperature and pH [36,38].

Class II bacteriocins such as plantaricin from *Lactobacillus plantarum*, may be either chromosomally or plasmid-encoded. Plantaricin 423 is plasmid encoded, whereas plantaricin ST31 is chromosomally encoded. However, where more than one bacteriocin is produced, the bacterioins can be both plasmid and chromosomally encoded. Genes encoding Class II bacteriocin production are organized within operon clusters and normally consist of a structural gene encoding the prepeptide, and dedicated immunity gene, an ABC-transporter gene for translocation through the membrane, and an accessory protein gene for exporting the bacteriocin. Occasionally, regulatory genes are also present [13,39]. The majority of the Class II bacteriocins are biosynthesized as an inactive prepeptide carrying an N-terminal leader peptide and a distinctive double-glycine proteolytic processing site, whereas Class IIc bacteriocins have a sec-type N-terminal signal sequence and are processed and secreted via the general secretary pathway. Except pediocin PA, onother Class II bacteriocin is plantaricin 423, which is plamid encoded. The plantaricin 423 encoding region has a similar operon structure to pediocin PA-1, with four ORFs (*plaABCD*) encoding the structural genes, immunity gene, accessory protein and ABC transporter. The *plaC* and *plaD* genes of plantaricin 423 have a 99% homology with *pedC* and *pedD* of pediocin PA-1. In addition, Class II bacteriocins also produce an induction factor that activates transcription of the regulated genes. This induction factor forms part of the signal transduction system responsible for biosynthesis of class II bacteriocins [13,39].

Bacteriocin-producing strains protect themselves from the inimical action of their own bacteriocin by producing specific immunity proteins. Genes encoding these proteins are in close genetic proximity to other bacteriocin structural and processing genes [13]. The immunity gene and the structural bacteriocin gene are most likely located on the same operon. Both the *Lan I* and the *Lan EFG* (multicomponent ABC transporter) systems have been described for LAB. The *Lan I* protein, in particular, provides immunity to producer cells by preventing pore formation by the bacteriocin molecules that have inserted into the membrane by pushing them back, thereby keeping the concentration of bacteriocin in the membrane under control [13,27].

The nature and composition of microbial growth media affect the amount of bacteriocin production. In a study by Goh and Phillip [40], a bacteriocin called weisellicin was produced from *Weisella confusa* using MRS medium. The bacteriocin started forming after 8 h of incubation and reached optimum production in 18 h. The antimicrobial activity of the bacteriocin was sustained between 18 and 24 h of growth but started to decrease after 28 h. Among 10 commercial media used, MRS gave the highest bacteriocin activity followed by LAPTg and M17 supplemented with different carbohydrates. Both temperature and pH have to be optimized for bacteriocin production.

Elayaraja et al. [41] found that 35 °C and pH 6.0 were ideal for the production of bacteriocin from *Lactobacillus murinus* AU06 isolated from marine sediments. Similarly, optimum conditions for bacteriocin production by *Lactobacillus acidophilus* were pH 6.0, 34 °C, with 4% phenyl acetamide [34]. In an earlier study, best conditions for bacteriocin production by *Lactobacillus plantarum* LPCO10 were obtained with temperatures ranging from 22 to 27 °C, NaCl concentration from 2.3 to 2.5%, and an inoculum size ranging from 10^7.3^ to 10^7.4^ CFU/ml, while fixing the glucose concentration at 2%, with no aeration of the culture [42]. Optimized conditions for bacteriocin production by *Lactobacillus viridescence* NICM 2169 were growth in MRS broth with pH 7.0, incubated at 37 °C for 48 h [43].

The LAB isolated from fermented meat serve as a source of novel bacteriocins. A strain of *Pediococcus acidilactici* LAB 5 produced the highest amount of bacteriocin when tryptone glucose yeast extract, Tween 80 and buffer medium were used and incubated at 37 °C for 24 h [44]. Optimized conditions for another isolate from fermented meat, *Lactobacillus sakei* subsp. *sakei* 2a, using Response Surface Methodology, were MRS broth supplemented with 5.5 g/L glucose and 1.05% Tween 20 at pH 6.28, and incubation at 25 °C [35]. Supplementation resulted in production of twice as much bacteriocin than commercial MRS broths under the same conditions [45].

Meanwhile, Onwuakor et al. [46] found that the optimum bacteriocin production by four *Lactobacillus* species, viz. *L. lactis*, *L. fermentum*, *L. casei*, and *L. plantarum*, that are active against *Salmonella typhimurium* (ATCC 14028), occurred when incubated at 30 to 35 °C, pH 5.0–6.0 and 2% NaCl for 72 h. When a resting cell system was developed for bacteriocin Lac-B23 production from *Lactobacillus paracasei* J23, incubated at 37 °C for 20 h, both cysteine and glycine stimulated bacteriocin production. Glycerol and pyruvic acid increased bacteriocin production at concentrations of 1% and 30 g/L, respectively. Interestingly, in this case, bacteriocin was found to act as an inducer of its own biosynthesis when added into the culture medium [47].

## 4. Urinary Tract Pathogens and Their Health Effects

Urinary tract infection (UTI) is a global clinical problem that is affecting people of all ages, commonly associated with inflammation of the urinary system, including the kidney, bladder, and urethra [48,49,50]. It is a cause for both nosocomial disease and community acquired infections. UTI ranks as the most common bacterial infection in humans, especially female adults; and is a challenging global health problem, especially in developing countries, and in parts of Europe and North America [49,50]. UTI is usually associated with urologic complications and can result in end-stage renal failure or hypertension if continued, and is associated with a high rate of morbidity and mortality [49]. *Escherichia coli* and Staphylococci constitute the normal microbiota of the human intestinal tract, but they cause infections when they invade the urinary system. UTI represents more than 30% of acute care cases in hospitals [50]. Common microorganisms implicated in UTI include *Candida albicans*, *Staphylococcus aureus*, *Methicillin Resistant Staphylococcus Aureus (MRSA)*, *Enterococcus* spp. (including *E. faecalis*), *Escherichia coli*, *Klebsiella pneumoniae*, *Proteus* spp., and *Pseudomonas aeruginosa* [49,50,51].

The best strategy to prevent UTI is maintaining proper hygiene of the genital area [40]. Genital and bladder infection arise due to the depletion or disturbance of the normal urogenital microbiota; particularly *Lactobacillus* species, which is most prevalent in healthy humans. Bacteria isolated from UTI patients are steadily increasing in their level of resistance to commonly used antibiotics, including ampicillin, trimethoprim-sulphamethozole (TMP-SMX) or co-trimoxazole and the quinolones, with most uropathogens being multi-drug resistant [49]. In a recent study by Mitiku [52], all isolates from patients showed a high rate of resistance to ampicillin, tetracycline, penicillin, vancomycin, cloxacillin, and amoxicillin, with the majority of them sensitive to ciprofloxin, ceftriaxone, nitrofurantoin, and norfloxacin. In this study, *E. coli* was the most prominent bacterial isolate, with increased prevalence in adult, female and married patients. As the antibiotic resistance phenomenon among bacterial pathogens evolves, routine surveillance and monitoring studies are essential to provide knowledge to physicians on the updated and most effective empirical treatment for UTIs. This growing problem of resistance of pathogenic bacteria to antibiotics requires the development of alternative strategies to prevent and treat UTI. The most recent strategies include the novel applications of probiotics, prebiotics and immuno-stimulants [45,53,54].

## 5. Activity of LAB against Uropathogens

The urogenital microbiota of healthy women comprises approximately 50 different species of microorganisms, which vary in composition according to reproductive stages and exposure to antibiotics and spermicides. Infection in the bladder and vagina arises when pathogens originating from the intestinal tract dominate the urogenital microbiota, with the concomitant depletion of indigenous organisms [55,56]. Since these infectious bacteria originate from the colon, consumption of lactic acid bacteria can serve as a source of beneficial microbiota. Complete and partial in vitro inhibition of uropathogens by LAB species has been documented by several researchers (Table 2), suggesting that lactobacilli strains vary in their ability to prevent colonization of uroepithelial cells by pathogens [12,57,58]. Nevertheless, inhibition of uropathogens by LAB is gaining prominence due to the increasing level of antibiotic resistance by a number of uropathogens. Although bacteriocins may not always be the agents responsible for the antimicrobial activity against uropathogens, they may be important in the colonization of the urogenital tract by LAB. Lactic acid and hydrogen peroxide are some of the metabolites LAB can use to control uropathogens.

Evidence suggests that LAB can inhibit the growth and attachment of uropathogens to uroepithelial cells under in vitro conditions [49,64]. Bacteriocin-producing *Lactobacillus fermentum* isolated from human breast milk demonstrated a broad spectrum of antimicrobial activity against uropathogens, including *Staphylococcus aureus*, *MRSA*, *Proteus* spp., *Enterococcus* spp., *Pseudomonas aeruginosa*, *Escherichia coli* and *Klebsiella pneumoniae* [51]. In an earlier study, *Lactobacillus acidophilus* CRL 1259 of human origin inhibited the growth of uropathogenic *E. coli*, prompting the inclusion of this strain in probiotic products for vaginal application [66]. In addition, vaginal *Lactobacillus* strains isolated from healthy women in the fertility age, inhibited the growth of uropathogens, especially *E. coli* through lactic acid production with or without other inhibitory substances [63].

A study by Ramasamy and Suyambulingam [59] confirmed bacteriocins of *Lactobacillus acidophilus* JCM1132 and *L. fermentum* BCS25 as potential broad-spectrum antibiotics against uropathogens, including *Streptococcus* spp., *E coli*, *Bacillus subtilis*, and *Pseudomonas* spp. Similarly, *Lactobacillus acidophilus* exhibited significant activity against multidrug-resistant isolates of *Pseudomonas aeruginosa* [60]. Elsewhere, LAB isolated from food samples exhibited excellent probiotic potentials against bacterial and fungal human pathogens, suggesting their use in the development of new pharmaceutical products for better public health [57].

Lim et al. [67] observed that three LAB isolates demonstrated major antimicrobial activity against all the uropathogens that were tested in their study. *Lactobacillus* strains inhibited the growth of uropathogenic *Neisseria gonorrhoea* and the yeast *Candida albicans*, when tested using the agar overlay method [64]. *Lactobacillus* strains isolated from fruits and vegetables displayed antimicrobial activity against six out of seven (85.7%) antibiotic-resistant uropathogens [50]. These two independent studies suggest that some LAB strains are potential candidates for the development of probiotics applicable in urinary tract disease treatment and prevention. Uropathogenic *Candida* spp. are capable of forming biofilms as they infect patients. In a study conducted by Bulgasem et al. [62], cell free supernatant of *Lactobacillus vurvatus* HH significantly reduced biofilm formation by *C. glabrata* ATCC2001 by 79.4% and *C. albicans* ATCC14053 by 61.1%. These results demonstrated that cell free supernatant of LAB can decrease the biofilm formation by *Candida* spp. and can be used in the prevention and treatment of candidiasis. 

Butler et al. [61] evaluated the protective and anti-inflamatory roles of the probiotic *Lactobacillus crispatus* strain CTV-05 in vitro. *L. crispatus* CTV-05 exhibited no cytotoxicity to vaginal epithelial cells compared to non-infected controls and provided significant protection against uropathogenic *E. coli* infection (*p* < 0.05), and creating no pro-inflamatory response in vitro, with no significant increase of IL-1β or IL-6. This established the potential of *Lactobacillus crispatus* strain CTV-05 as a probiotic treatment to reduce the risk of recurrent UTI. Most recently, four lactobacilli strains, *L. animalis* LMEM6, *L. plantarum* LMEM7, *L. acidophilus* LMEM8 and *L. rhamnosus* LMEM9, isolated from curd samples inhibited indicator bacteria by agar-well diffusion and agar overlay methods. The average growth inhibitory activity of the lactobacilli ranged from 233.34 ± 45.54–280.56 ± 83.67 AU/mL, indicating their potential as candidate probiotics and bio-therapeutics against human pathogens, including uropathogens.

Liu et al. [58] evaluated the in vitro and in vivo antimicrobial activity of selected LAB against uropathogenic *E. coli* (UPEC) for the prevention and treatment of UTIs. Seven LAB strains and their products exhibited potent inhibition against UPEC, and adhered strongly to the uroepithelium SV-HUC-1 cell line. In addition, the growth of UPEC strains was strongly inhibited by a co-culture of LAB and the probiotic products in urine. The addition of LAB strains and probiotic products in UPEC-induced SV-HUC-1 cells significantly decreased levels of IL-6, IL-8 and lactic acid dehydrogenase. The outcomes of this study suggest the application of probiotic supplementation as adjuvant therapy for the treatment and amelioration of bacterial-induced UTI.

The application of LAB in treating and preventing urogenital infection by oral intake of probiotic organisms is attractive to both patients and caregivers as it allows for the self-administration of therapy [68]. Probiotic LAB are no magic bullets, but as Jassawala [69] argues, they help restore and maintain urogenital and intestinal health, suggesting that a daily intake of scientifically selected strains would provide a safe and effective means of regulating the fluctuating vaginal microbiota, thereby lowering the risk of infection in women.

Earlier in vivo and clinical studies on the application of LAB in the treatment and prevention of uropathogens were also conducted by Johansson et al. [70], where strains of *Lactobacillus plantarum*, *L. reuteri*, *L. casei* subsp. *rhamnosus*, and *L. agilis* were recovered 11 days after they had implanted in the intestinal mucosa, though *L. plantarum* spp. were dominant. However, these authors did not confirm whether these strains subsequently colonized the female urogenital tract. Instead, Reid et al. [71] provided clinical evidence for the delivery of *Lactobacillus rhamnosus* GR-1 and *Lactobacillus reuteri* RC-14 (formerly called *Lactobacillus fermentum* RC-14) to the vagina following oral intake. Additional evidence from in vivo and clinical studies are included in Table 3. Administration of these strains was able to resolve bacterial vaginosis within one week of therapy. However, as argued by Slačanac et al. [72] the incorporation of these strains to food, process parameters and the processing of their fermentative products warrant further investigation. More in vivo and clinical studies are needed to validate the role of dietary LAB in the prevention of UTIs, and this requires placebo-controlled, blinded studies using oral route, with sufficiently large study groups.

Oral intake of LAB must be done in a way that ensures cell viability through the harsh conditions in the stomach and successful delivery to the intestinal tract. According to a review by Yadav et al. [77], microencapsulation of LAB with a gastro-resistant material has been used in the pharmaceutical industry among other applications, to efficiently deliver living cells to the target sites. Alginate is the most commonly used material for probiotics encapsulation. Other materials used for this purpose include gelatin, chitosan, carrageenan, whey proteins, cellulose acetate phthalate, locust bean gum and starches. Tablet formulations can also be done for LAB to prevent their degradation in the stomach. Other proprietary and patented delivery technologies include Stomach Acid Resistance (STAR) technique, which is an enteric coating process to protect probiotic LAB in the stomach by preventing solubilization by gastric juices; PROBIOCAP prevents destruction of LAB such as *L. rhamnosus*, *L. acidophilus* in the stomach, allowing release only in the intestine based on pH; BIO-TRACT is a Controlled Delivery Technology (CDT) that pertains manufacture of tablets and capsules, which release active ingredients either at a constant rate or at precisely timed intervals. Through this technology, *L. acidophilus* can be released in the upper small intestine, and bifidobacteria in the lower large intestine. Other product formulations include chewable tablets, sachets, and oil suspensions. Supplement formulations may contain other active components such as vitamins and prebiotics [78,79]. Preventive effects of LAB have been demonstrated in various urogenital infection cases, and the antimicrobial activities of LAB against gastrointestinal and uropathogens. LAB have great potential, particularly in the era of increasing threat posed by antibiotic-resistant microorganisms [80].

There has been considerable interest in recent decades regarding the application of LAB in the management of gastrointestinal disorders and urinary tract infections (UTIs). The incorporation of LAB in functional dairy products such as ice-cream, sour cream, cheese, yoghurt, powdered milk, and frozen desserts, renders probiotic LAB accessible to people of all age groups [81]. New trends of probiotic applications include oral vaccine development, anti-carcinogenic, anti-diabetic, anti-allergic and anti-inflammatory activities and as genetically modified probiotics for therapeutic purposes [82].

## 6. Conclusions

Lactic acid bacteria and their bacteriocins have emerged as great alternatives to chemicals and antibiotics in the fields of food technology and some have demonstrated antimicrobial activities against uropathogens and gastrointestinal pathogens. In the current era of antibiotic resistance, LAB and bacteriocins may be the only remedy for some clinical cases, although they may be required to be used in combination with low dosages of antibiotics in some instances. Novel applications of LAB and bacteriocins are steadily increasing, with prospects of more fascinating roles to be played by these agents in future, such as in anti-quorum sensing strategies and site-specific drug delivery. However, in view of the risk of possible horizontal transfer of antibiotic resistance genes through LAB, the choice of isolates must follow strict guidelines in addition to the antimicrobial actions. Therefore, more focused research studies need to be conducted to include in vitro and in vivo analyses, animal model studies and human trials, in order to validate health claims, and to ensure the safety and efficacy of LAB and their bacteriocins for clinical applications.

## Figures and Tables

**Table 1:** Classes and properties of bacteriocins from lactic acid bacteria.

Class	Typical Producing Species	Properties	Examples (References)
I	*Lactobacillus lactis subsp. lactis*	Contain unique amino acids, i.e., lanthionine and methyllanthionine; <5 kDa	nisin, lactocin, mersacidin [11,13,34,35]
IIa	*Leuconostoc gelidum*	Heat stable, non-modified, cationic, hydrophobic peptides; contain a double–glycine leader peptide; pediocin-like peptides; <10 kDa	pediocin PA1, sakicin A, leucocin A [13,27,36,37]
IIb	*Enterococcus faecium*	Require synergy of two complementary peptides; mostly cationic peptides	lactococcin G, plantaricin A, enterocin X [29,30]
IIc	*Lactobacillus acidophilus*	Affect membrane permeability and cell wall formation	acidocin B, entereocin P, reuterin 6 [35]
III	*Lactobacillus helveticus*	Heat-labile; large molecular mass peptides; >30 kDa	lysostaphin, enterolysin A, helveticin J [11,13,29,34]

**Table 2:** In vitro studies of antimicrobial activities of LAB isolates against uropathogens.

LAB species	Target pathogens	References
*Lactobacillus fermentum*	*Staphylococcus aureus*; MRSA; *Proteus* spp.; *Enterococcus* spp.; *Escherichia coli*; *Pseudomonas aeruginosa*; *Klebsiella pneumoniae*	[51,59]
*L. acidophilus*	Uropathogenic *E*. *coli*; *P. aeruginosa*	[60]
*L. crispatus*	Uropathogenic *E*. *coli*	[61]
*L. vurvatus*	*C. albicans*	[62]
*Lactobacillus* spp.	*E. coli*; *Neisseria gonorrhoea*; *Candida albicans*; *Salmonella* spp.; *S. aureus*; *S. typhimurium*; *B. cereus*; *P. aeruginosa*	[63,64,65]

**Table 3:** In vivo and clinical studies of antimicrobial activities of LAB isolates against uropathogens.

LAB species	Uropathogens/Condition	References
*L. rhamnosus*, *L. acidophilus*	*Gardnerella vaginalis*	[73]
*L. rhamnosus* GR-1, *L. reuteri* RC-14	*Candida albicans* Uropathogenic *E. coli*	[71]
*L. crispatus*	Bacterial vaginosis	[74]
*L. gasseri* CRL 1509, *L. salivarius* CRL 1328	*Streptococcus agalactiae*	[75]
*L. acidophilus*	Uropathogenic *E. coli*	[76]
